# Genome Sequences of Uncommon Shiga Toxin-Producing Escherichia coli Serotypes

**DOI:** 10.1128/MRA.01496-19

**Published:** 2020-03-05

**Authors:** Baha Abdalhamid, Emily L. Mccutchen, Peter C. Iwen, Joao Carlos Gomes-Neto, Andrew K. Benson, Steven H. Hinrichs

**Affiliations:** a Department of Pathology and Microbiology, University of Nebraska Medical Center, Omaha, Nebraska, USA; b Nebraska Public Health Laboratory, University of Nebraska Medical Center, Omaha, Nebraska, USA; c Department of Food Science and Technology, University of Nebraska, Lincoln, Nebraska, USA; University of Maryland School of Medicine

## Abstract

Shiga toxin-producing Escherichia coli (STEC) is a foodborne disease with worldwide outbreaks. STEC serotypes O157, O26, O45, O103, O111, O121, and O145 cause the most outbreaks. There is little published information regarding the other serotypes. We report the draft genome sequences for 11 uncommon STEC serotypes from Nebraska.

## ANNOUNCEMENT

Shiga toxin-producing Escherichia coli (STEC) can cause hemorrhagic colitis and a life-threatening hemolytic uremic syndrome (1, 2). STEC causes outbreaks that are a worldwide public health concern (2–4). The 6 non-O157 STEC serotypes, O26, O45, O103, O111, O121, and O145, in addition to the STEC O157 serotype are responsible for an overwhelming majority of outbreaks (5, 6). Information regarding the other STEC serotypes is scarce (4). We present in this study draft genome sequences for 11 different uncommon STEC serotypes collected from Nebraska.

From November 2017 to February 2019, 120 STEC isolates were collected from different laboratories in Nebraska following routine stool culture for original isolation. Stool samples were subcultured on blood, MacConkey agar, Hektoen enteric agar, and MacConkey agar with sorbitol agar medium and were incubated overnight at 37°C. Isolates were then transported to the Nebraska Public Health Laboratory (NPHL) for further characterization. At NPHL, isolates were grown overnight on blood agar plates at 37°C. A MagNA Pure compact nucleic acid isolation kit I (Roche Diagnostics, IN, USA) was used to extract genomic DNA from overnight-grown isolates using a MagNA Pure compact instrument (Roche Diagnostics) following the manufacturer’s instructions. A NanoDrop 2000 UV-visible (UV-Vis) spectrophotometer (Thermo Fisher, MA, USA) and a Qubit 3.0 fluorometer (Invitrogen, CA, USA) were used to determine DNA measures qualitatively and quantitatively, respectively.

Bacterial genomic DNA libraries were constructed using the Nextera XT library prep kit (Illumina, CA, USA) as recommended by the manufacturers. Whole-genome sequencing (WGS) was performed using the Illumina MiSeq platform (Illumina, CA, USA) to generate 300-bp paired-end reads. A rate of clusters passing the filter of >80%, a Phred quality score (QS30) of >75%, and a cluster density of 600 to 1,300 were used as parameters to assess the quality of the run. FastQC 0.10, Trimmomatic 0.33, SPAdes 3.12, BBMap 38.06, and QUAST 4.1 (7–10) were used for assessing the quality of sequence reads, trimming, *de novo* assembly, purging of contigs less than 200 bp, and determining the quality of the *de novo* assembled genomes, respectively (Table 1). These bioinformatics tools were used on GitHub. Default parameters were used for all software unless otherwise specified. The NCBI Prokaryotic Genome Annotation Pipeline (PGAP) 4.8 (https://www.ncbi.nlm.nih.gov/genome/annotation_prok/) was used to annotate the draft genome of each strain (11).

**TABLE 1:** Summary characteristics of whole-genome sequencing of uncommon serotypes of Shiga toxin-producing E. coli

Strain code^*a*^	Serotype^*b*^	ST^*c*^	Genome size (bp)	No. of contigs	% GC content	*N*_50_ (bp)	No. of reads	Genome coverage (×)	SRA accession no.	GenBank accession no.
STEC_170836	O185:H28	ST-517	5,162,389	112	50.64	15,2751	1,128,853	56.44	SRX6741578	VZEL00000000
STEC_180018	O69:H11	ST-14	5,399,941	260	50.38	131,480	1,148,343	57.42	SRX6741577	VZEP00000000
STEC_180309	O118/O151:H16	ST-73	5,478,013	293	50.42	92,792	968,438	48.42	SRX6741575	VZFK00000000
STEC_180427	O118/O151:H16	ST-335	5,416,226	310	50.41	90,305	1,067,885	53.39	SRX6741582	VZEC00000000
STEC_180452	O55:H7	ST-131	5,359,179	151	50.42	311,454	2,824,168	141.21	SRX6741581	VZDR00000000
STEC_180473	O123/O186:H2	ST-658	5,493,863	277	50.42	137,641	1,534,681	76.92	SRX6741580	VZFY00000000
STEC_180505	O118/O151:H16	ST-21	5,419,695	314	50.43	89,301	1,072,233	53.61	SRX6741579	VZGA00000000
STEC_180563	O151:H16	ST-17	5,567,883	310	50.37	82,954	1,129,349	56.47	SRX6741584	VZGH00000000
STEC_180607	O123:H2	ST-21	5,330,230	271	50.58	136,012	1,408,908	70.45	SRX6741574	VZGR00000000
STEC_180727	O88:H25	ST-21	5,016,338	136	50.64	168,851	1,929,340	96.47	SRX6741572	VZDY00000000
STEC_180784	071:H49	ST-21	5,477,716	348	50.44	104,253	1,349,778	67.49	SRX6741571	VZEA00000000

a All strains carried *stx*_1_. STEC_170836 carried both *stx*_1_ and *stx*_2_.

b Serotypes are predicted based on the whole-genome sequencing.

c ST, sequence type determined by MLST.

KmerFinder 3.1 and SeroTypeFinder 2.0 were used to determine organism identification and serotypes, respectively, for the STEC isolates (12, 13). In addition, multilocus sequencing types (MLSTs) were determined using MLST 2.0 (Table 1) (14).

The average genome size of the sequenced isolates was 5,374,679 bp, with a range of 5,016,338 to 5,567,883 bp. The average GC content was 50.5%, with a range of 50.37% to 50.64%. The average number of contigs was 253, with a low of 112 contigs and a high of 348 contigs. The highest *N*_50_ value was 311,454 bp, while the lowest *N*_50_ value was 82,954 bp, with the average being 136,163 bp.

The data presented in this study will provide the genetic basis for a more detailed analysis of virulence factors, antimicrobial resistance determinants, and phylogenetic relatedness among these strains.

### Data availability.

PRJNA527109 is the BioProject number for these genomic sequences at NCBI. The numbers of contigs, *N*_50_ values, serotypes, MLSTs, total number of reads generated for each isolate, genome coverage, GenBank accession numbers, and Sequence Read Archive (SRA) accession numbers are provided in Table 1.
